# Coronary calcification score is higher in type 2 diabetic patients with cardiovascular autonomic neuropathy

**DOI:** 10.1590/S1516-31802007000200013

**Published:** 2007-03-04

**Authors:** Luiz Clemente de Souza Pereira Rolim, Paulo Henrique Rezende, João Roberto Sá, Fabio Nasri, Romeu Meneghello, Antonio Roberto Chacra, Sérgio Atala Dib

An association between cardiovascular autonomic neuropathy (CAN) and mortality among individuals with diabetes is known.^1,2^ However, the pathophysiology of this association is still under discussion. Silent myocardial ischemia is common in diabetics with CAN. Both parasympathetic and sympathetic pathways may be involved.^3^ There are data suggesting that the extent of coronary calcification and the development of ischemic heart disease seem to be closely related to diabetic complications.^4^ The present study was aimed at investigating the degree of coronary artery calcification in type 2 diabetes mellitus individuals (T2DMs) with CAN (WCAN; n = 9) and without CAN (WOCAN; n = 9).

A pilot study was conducted on 18 T2DMs, using the following inclusion criteria: diabetes diagnosed more than 10 years earlier (TDDM), normal resting electrocardiogram, non-smoking and being asymptomatic for coronary artery disease. The exclusion criteria were drug use for hyperlipidemia, glomerular filtration rate lower than 50 ml/min, congestive heart failure and history of stroke. Informed written consent was obtained from all patients and the Ethics Board of Universidade Federal de São Paulo had previously approved the protocol.

Fasting serum C-peptide (normal value): 0.36-3.4 ng/ml), HbA1c (A1c Hemoglobin; normal value: 3.4-6.8%), lipids and urinary albumin/creatinine ratio were measured by standard laboratory tests. Arterial hypertension and electrocardiogram (EKG) (Marquette MAC500) were also evaluated. Electron beam computed tomography imaging was performed using an ultrafast scanner (C-150 Imatron) and the coronary calcium score (CaS) was calculated using Agatston’s method.^5^ The CAN diagnosis was based on two or more abnormal cardiovascular autonomic tests on two different occasions. These tests were, firstly, the heart rate variability (HRV) at six deep breaths per minute (mean); secondly the 30:15 ratio appraised for the relationship between the longest RR interval of EKG around the thirtieth beat and the shortest interval around the fifteenth beat after the patient was standing; and finally, the orthostatic hypotension that was present when systolic blood pressure decreased by at least 20 mmHg in the third minute of standing.

The differences between WCAN and WOCAN were analyzed using the Mann-Whitney or Student t test. The significance level chosen was 0.05 for all statistical tests.

The two groups did not differ in relation to: sex (males: 44.4% versus 55.5%), age (54.7 ± 5.5 versus 59.2 ± 3.9 years), TDDM (14.4 ± 4.0 versus 13.7 ± 2.9 years), body mass index (28.2 ± 2.9 versus 27.6 ± 4.0 kg/m^2^), hypertension (88.9% versus 100%), albuminuria (55.6% versus 55.6%), high-density lipoprotein (1.36 ± 0.35 versus 1.06 ± 0.29 mmol/l), low-density lipoprotein (3.30 ± 0.81 versus 3.36 ± 1.14 mmol/l), triglyceride (1.65 ± 0.83 versus 2.11 ± 0.48 mmol/l), basal C-peptide (0.59 ± 0.33 versus 0.74 ± 0.73 nmol/l) and HbA1c (9.0 ± 1.3 versus 8.2 ± 1.5%). However, the coronary CaS in WCAN (565.0; 225.9 - 805.5) was significantly higher (p = 0.01) than in WOCAN (74.5; 6.6 - 221.8).

Therefore, despite the small number of individuals in this pilot study, the results suggest that even in T2DMs with similar clinical and metabolic characteristics, coronary calcification is more prevalent when CAN is present. Some points regarding this association had been shown in other studies.^6,7^ However, greater numbers of subjects and long-term follow-up would help in determining the true association between CaS and CAN and its value in predicting coronary heart disease events in Type 2 diabetes.
